# Detection of CD39 and a Highly Glycosylated Isoform of Soluble CD73 in the Plasma of Patients with Cervical Cancer: Correlation with Disease Progression

**DOI:** 10.1155/2020/1678780

**Published:** 2020-12-07

**Authors:** Ricardo Muñóz-Godínez, María de Lourdes Mora-García, Benny Weiss-Steider, Juan José Montesinos-Montesinos, Adriana del Carmen Aguilar-Lemarroy, Rosario García-Rocha, Jorge Hernández-Montes, Christian Azucena Don-López, Luis Roberto Ávila-Ibarra, Daniela Berenice Torres-Pineda, Gabriela Molina-Castillo, Rommel Chacón-Salinas, Luis Vallejo-Castillo, Sonia Mayra Pérez-Tapia, Alberto Monroy-García

**Affiliations:** ^1^Laboratorio de Inmunología y Cáncer, Unidad de Investigación Médica en Enfermedades Oncológicas, CMN SXXI, Instituto Mexicano del Seguro Social, Ciudad de México, Mexico; ^2^Programa de Posgrado en Ciencias Biológicas, UNAM, Ciudad de México, Mexico; ^3^Laboratorio de Inmunobiología, UIDCC-UMIEZ, FES-Zaragoza, UNAM, Ciudad de México, Mexico; ^4^Laboratorio de Células Troncales Mesenquimales, Unidad de Investigación Médica en Enfermedades Oncológicas, CMN SXXI, Instituto Mexicano del Seguro Social, Ciudad de México, Mexico; ^5^Centro de Investigación Biomédica de Occidente División de Inmunología Sierra Mojada, No. 800, Col. Independencia, C.P. 44340 Guadalajara, Jalisco, Mexico; ^6^Unidad de Desarrollo e Investigación en Bioprocesos (UDIBI), Instituto Politécnico Nacional, Ciudad de México, Mexico; ^7^Departamento de Inmunología, Escuela Nacional de Ciencias Biológicas, Instituto Politécnico Nacional, ENCB-IPN, Ciudad de México, Mexico; ^8^Departamento de Farmacología, Centro de Investigación y de Estudios Avanzados del IPN (Cinvestav-IPN), Ciudad de México, Mexico; ^9^Laboratorio Nacional para Servicios Especializados de Investigacioón, Desarrollo e Innovación (I + D + i) para Farmoquímicos y Biotecnológicos (LANSEIDI-FarBiotec-CONACyT), Escuela Nacional de Ciencias Biológicas, Instituto Politécnico Nacional, Mexico City, Mexico

## Abstract

Persistent infection with high-risk human papillomavirus (HR-HPV) is the main factor in the development of cervical cancer (CC). The presence of immunosuppressive factors plays an important role in the development of this type of cancer. To determine whether CD39 and CD73, which participate in the production of immunosuppressive adenosine (Ado), are involved in the progression of CC, we compared the concentrations and hydrolytic activity of these ectonucleotidases in platelet-free plasma (PFP) samples between patients with low-grade squamous intraepithelial lesions (LSILs) (*n* = 18), high-grade squamous intraepithelial lesions (HSILs) (*n* = 12), and CC (*n* = 19) and normal donors (NDs) (*n* = 15). The concentrations of CD39 and CD73 in PFP increased with disease progression (*r* = 0.5929, *p* < 0.001). The PFP of patients with HSILs or CC showed the highest concentrations of CD39 (2.3 and 2.2 times that of the NDs, respectively) and CD73 (1.7 and 2.68 times that of the NDs, respectively), which were associated with a high capacity to generate Ado from the hydrolysis of adenosine diphosphate (ADP) and adenosine monophosphate (AMP). The addition of POM-1 and APCP, specific inhibitors of CD39 and CD73, respectively, inhibited the ADPase and AMPase activity of PFP by more than 90%. A high level of the 90 kD isoform of CD73 was detected in the PFP of patients with HSILs or CC. Digestion with endoglycosidase H and N-glycanase generated CD73 with weights of approximately 90 kD, 85 kD, 80 kD, and 70 kD. In addition, the levels of transforming grow factor-*β* (TGF-*β*) in the PFPs of patients with LSIL, HSIL and CC positively correlated with those of CD39 (*r* = 0.4432, *p* < 0.001) and CD73 (*r* = 0.5786, *p* < 0.001). These results suggest that persistent infection by HR-HPV and the concomitant production of TGF-*β* promote the expression of CD39 and CD73 to favor CC progression through Ado generation.

## 1. Introduction

Cervical cancer (CC) is the fourth most common type of cancer in women and represents a major public health problem worldwide because more than 500,000 new cases and approximately 250,000 deaths are reported each year, more than 80% of which occur in developing countries [1]. Persistent infection by high-risk human papillomavirus (HR-HPV) is the main factor in the development of low-grade squamous intraepithelial lesions (LSILs), which can progress to high-grade lesions (HSILs) and eventually to CC [2]. To date, more than 200 HPV genotypes have been identified, and HPV-16, HPV-18, HPV-31, HPV-33, HPV-35, HPV-39, HPV-45, HPV-51, HPV-52, HPV-56, and HPV-58, which are considered HR-HPVs, are associated with anogenital cancer [3, 4]. Although the immune response against HPV antigens eliminates most infections and precursor lesions, some women exposed to HR-HPV will develop cancer, suggesting that other risk factors may be involved [5]. A growing number of studies have suggested that immunoregulation may play an important role in the development of CC. Recently, the adenosinergic pathway has been proposed to play an important role in essential signaling related to tumor growth, immunosuppression, and metastasis in cancer [6, 7]. In this pathway, the nucleotides adenosine triphosphate (ATP) and adenosine diphosphate (ADP) increase to high concentrations greater than 50 *μ*M in response to stress signals, such as hypoxia, damage, and inflammation in the tumor microenvironment (TME), and are hydrolyzed by the ectoenzyme CD39 (ectonucleoside triphosphate diphosphohydrolase-1, ENTPD1; EC 3.6.1.5) to AMP and subsequently to adenosine (Ado) by the activity of 5′-ectonucleotidase (CD73, EC 3.1.3.5) [8, 9]. Most Ado extracellular signaling activities are mediated by four receptor subtypes (adenosine receptors (ARs): A1R, A2AR, A2BR, and A3R) coupled to G proteins in the target cell membrane [10, 11]. In the TME, high concentrations of Ado (10–100 *μ*M) are generated through adenosinergic activity and exert important effects on the host, such as an immunosuppressive effect on CD8^+^ cytotoxic T lymphocytes (CTLs), NK cells, B cells, and dendritic cells by interacting with the high-affinity A2AR [12]. In addition, Ado can promote tumor growth by inducing the proliferation, invasion, and metastasis of tumor cells, mainly through its interaction with A1R, A2AR, and A2BR [13]. Upregulation of CD39 and CD73 in several types of tumors has been associated with a poor clinical prognosis [14, 15]. In addition, extracellular vesicles derived from squamous cell carcinoma of the head and neck [16], prostate cancer [17], neuroblastoma [18], and other types of neoplastic cells [19, 20] can generate an immunosuppressive environment through the generation of Ado from ATP hydrolysis. Likewise, the presence of high levels of CD73 in the plasma of cancer patients has been correlated with advanced stages of the disease [21–24], suggesting that extracellular adenosinergic activity may play an important role in the pathophysiology of cancer.

Recently, we provided evidence that cells obtained from cervical samples of patients with low-grade intraepithelial neoplasms (CINI) positive for HPV-16 showed higher concentrations of CD39 and CD73 than cells from samples of patients with CINI negative for HPV-16 and from normal donors (NDs). The solubilized cervical mucus of these patients also showed higher concentrations of soluble CD39 and CD73, which was associated with a greater capacity to produce Ado through the hydrolysis of ATP and AMP [25].

To determine whether CD39 and CD73 are involved in the development of CC, we analyzed the concentrations and hydrolytic activity of these ectonucleotidases in plasma samples from patients with LSILs, HSILs, or CC. For comparison, ND plasma samples were also analyzed.

## 2. Methods

### 2.1. Biological Material

Biological samples were obtained from women who attended early detection programs at the Gynecology and Obstetrics Hospital No. 4 of the Mexican Social Security Institute (*Instituto Mexicano del Seguro Social* (IMSS)), the gynecology service of the Oncology Hospital of CMN SXXI of the IMSS, Mexico City, Mexico, and the Western National Medical Center (CMNO-IMSS) in Guadalajara, Jalisco, Mexico, between April 2016 and May 2018 after signing the informed consent form endorsed by the local bioethics committee. Women without cervical lesions were diagnosed by conventional cytology (Papanicolaou stain) and colposcopy. In cases of precancerous lesions or CC, the diagnosis was confirmed by histopathology. The cytologies and biopsies were analyzed by the pathologists of each clinic and were classified according to the Bethesda System 2001 [26] as follows: negative for intraepithelial lesion or malignancy (NILM); cervical intraepithelial neoplasia grades I, II, and III (CINI, CINII, and CINIII, respectively); and invasive CC. To analyze the data, the lesions were grouped according to the classification of the squamous intraepithelial lesion (SIL), either low-grade SIL (LSIL, corresponding to the histological classification of CINI) or high-grade SIL (HSIL, corresponding to the histological classification of CINII and CINIII). Samples diagnosed as CC constituted the last group.

Cervical samples from women diagnosed with NILM were collected during gynecological examinations with a cytobrush (Cytobrush®, STERYLMEDICAL Co., Yangon, Myanmar) and then placed in transport medium (PreservCyt Solution; Hologic, Bedford, MA) to be stored at 4°C until DNA extraction. The samples were analyzed by conventional single-round polymerase chain reaction (PCR) to rule out the presence of HPV using the MY09/MY11 primers [27]; women who were consistently negative in clinical and molecular tests were considered NDs. As a positive control, DNA from the HeLa cell line (HPV-18^+^) was used as previously reported [28].

The cervical samples of women diagnosed with SILs or CC were subjected to molecular analysis by PCR using the LINEAR ARRAY® HPV kit (Roche Diagnostics, CA, USA) for genotyping of the 37 main types of HPV that infect the anogenital region (HPV-6, HPV-11, HPV-16, HPV-18, HPV-26, HPV-31, HPV-33, HPV-35, HPV-39, HPV-40, HPV-42, HPV-45, HPV-51, HPV-52, HPV-53, HPV-54, HPV-55 (HPV-44 subtype), HPV-56, HPV-58, HPV-59, HPV-61, HPV-62, HPV-64 (HPV-34 subtype), HPV-66, HPV-67, HPV-68, HPV-69, HPV-70, HPV-71, HPV-72, HPV-73 (MM9), HPV-81, HPV-82 (MM4), HPV-83 (MM7), HPV-84 (MM8), IS39 (HPV-84 variant), and HPV-89 (CP6108)). In each sample, the human beta-globin gene was amplified as an internal control. After the hybridization reaction, the strips were visually read against a reference guide. All procedures followed the manufacturer's instructions.

The peripheral blood samples used in this study were obtained from 15 NDs, 18 patients with LSILs, 12 patients with HSILs, and 19 patients with CC (Tables 1 and 2). The samples were collected in vacutainer tubes with an ACD anticoagulant (Becton Dickinson, USA) and centrifuged at 385 × *g* for 10 min to isolate the plasma. The blood plasma was centrifuged at 2422 × *g* for 10 min at 4°C to separate platelets as previously reported [29]. Soluble CD39 and CD73 were quantified in platelet-free plasma (PFP).

### 2.2. Detection and Quantification of Soluble CD39 and CD73 in PFP

CD39 and CD73 were detected in PFP by the enzyme-linked immunosorbent assay (ELISA). The data were interpolated in type curves using different concentrations (1-35 ng/ml) of human recombinant enzymes (rhCD39 and rhCD73, R&D Systems, Minneapolis, MN, USA) diluted in phosphate-buffered saline (PBS). PFP was diluted with PBS, and CD39 was detected in PFP diluted to 1 : 40,000 and CD73 to 1 : 25,000. Samples of 100 *μ*l of the different dilutions were placed in triplicate in 96-well flat-bottomed ELISA/radioimmunoassay plates (Corning Inc., USA). The plates were incubated for 1 h at 37°C and then overnight at 4°C. The plates were washed with a washing solution (PBS 0.1% Tween-20) and then incubated with a blocking solution (2% BSA *w*/*v* in PBS 0.1% Tween-20) for 2 h at 37°C. After washing, an anti-CD39 or anti-CD73 antibody (Novus Biologicals, USA) was added at a 1 : 1000 dilution in blocking solution and incubated for 2 h at 37°C. The plates were washed six times and incubated with the secondary goat anti-mouse or anti-rabbit IgG bound to alkaline phosphatase (Thermo Fisher Scientific, Waltham, MA, USA) in a 1 : 500 dilution in blocking solution and incubated for 2 h at 37°C. After eight washes, the substrate for alkaline phosphatase (Sigma-Aldrich, St. Louis, MO, USA) diluted in diethanolamine (Sigma-Aldrich, St. Louis, MO, USA) was added to 10% (pH 9.8). Finally, the reading was performed at a wavelength of 405 nm in an ELISA plate reader.

### 2.3. Hydrolytic Activity of Soluble CD39 and CD73

To determine the hydrolytic activity of CD39 and CD73 ectonucleotidases contained in the PFP, 5 *μ*l of each PFP sample was incubated in the presence of ADP or AMP at a final concentration of 5 mM. After 72 h of incubation, Ado production was evaluated. To inhibit the enzymatic activity of CD39 and CD73, the specific inhibitors polyoxotungstate sodium (POM-1, Sigma-Aldrich, St. Louis, MO, USA) and adenosine 5′-(*α*,*β*-methylene) diphosphate (APCP, Sigma-Aldrich), respectively, were used at a final concentration of 5 mM, as previously described [30]. The total volume of each reaction was 100 *μ*l. The amount of Ado produced by each sample incubated with ATP or AMP was evaluated through ultra-high-performance liquid chromatography (UPLC) after applying 25 *μ*l of each reaction to a chromatograph (UPLC Acquity, Waters Corporation, Milford, MA, USA) using a mobile phase composed of 0.5% acetonitrile, 5% methanol, and 94.5% sodium acetate (0.25 M and pH 6.3). Before reading, the samples were filtered through 3000 D Amicon filters (Millipore Corporation, USA). A standard Ado curve was prepared in Empower 3 (Waters Corporation, Milford, MA, USA) to evaluate the Ado concentrations in the different samples.

### 2.4. Quantification of TGF-*β*1

To quantify the TGF-*β*1 in PFP samples, the Quantikine human TGF-*β*1 ELISA kit (R&D Systems) was used according to the manufacturer's protocol.

### 2.5. Western Blot

To analyze the presence of CD73, samples of 3 *μ*l of each PFP or 20 ng of CD73 contained in the PFP (based on the rhCD73 type curve) were treated with Laemmli buffer and analyzed by 10% sodium dodecyl sulfate- (SDS-) polyacrylamide gel electrophoresis. Proteins were transferred to a nitrocellulose membrane (Amersham Protran, 0.2 *μ*m), which was incubated with blocking solution (TBS 0.1% Tween-20 and 5% BSA) for 60 min at room temperature. The membrane was washed twice with washing solution (TBS 0.1% Tween-20) and then incubated with anti-CD73 (Novus Biologicals, USA) at a 1 : 1000 dilution in blocking solution overnight at 5°C. After five washes, the membrane was incubated with the secondary antibody (HRP goat anti-rabbit) at a 1 : 1500 dilution with blocking solution for 1 h. After five washes, the presence of CD73 in the membrane was revealed in ChemiDoc (Life Science Research Bio-Rad, USA) using the Chemiluminescent Peroxidase Substrate (Sigma-Aldrich, USA).

### 2.6. Enzymatic Deglycosylation

To deglycosylate CD73 contained in PFP samples, these samples were incubated for 5 min at 37°C in the presence of 1.5 *μ*l of a denaturing solution (1 M *β*-mercaptoethanol and 2% SDS). Next, the endoglycanase H (Genzyme, Co., USA) and N-glycanase (Genzyme, Co., USA) enzymes were added to a final concentration of 0.05 U/ml and incubated for 18 h at 37°C. Last, the products of enzymatic digestion were analyzed by Western blot using the anti-CD73 antibody as described above.

### 2.7. Statistical Analysis

All numerical data are presented as the mean value ± standard error of the mean (SEM) of three independent experiments. The comparisons and correlations were evaluated by multivariate statistical analysis with GraphPad Prism version 7 (La Jolla, CA, USA).

## 3. Results

### 3.1. Characteristics of the Participants

The present study was carried out with 64 cervical and peripheral blood samples from women who attended early cancer detection programs at the Gynecology and Obstetrics Hospital No. 4 of the IMSS and the gynecology service of the Oncology Hospital of CMN SXXI of the IMSS, Mexico City, Mexico, and the Western National Medical Center (CMNO-IMSS) in Guadalajara, Jalisco, Mexico. According to the cytological and histopathological analysis, 15 samples came from ND women (Table 1), 18 LSIL patients, 12 HSIL patients, and 19 CC patients (Table 2). All samples and clinical data of the participants were taken after obtaining informed consent according to ethical requirements and confidentiality related to the sampling of humans in the institutions involved.

To rule out the presence of HPV infection in the samples of NILM women, DNA was obtained from the cervical samples for molecular analysis by PCR using the consensus oligonucleotides MY09 and MY11, which amplify a conserved fragment of 450 bp of the gene coding the L1 protein of the different HPV genotypes. As a positive control, DNA from the HeLa cell line (HPV-18^+^) was used (Supplementary Figure 1). Nineteen cervical samples from NILM women were analyzed, 15 of which were negative for HPV infection and were included in the study as ND samples. The average age of the NDs was 31.2 (range 22-41) years (Table 1), that of women with LSILs was 32.7 (range 21-43) years, that of women with HSILs was 36.5 (range 27-46) years, and that of women with CC was 47.68 (range 35-62) years (Table 2). The average numbers of sexual partners and pregnancies in the ND group were 2.26 (range 1-4) and 1 (range 0-3), respectively (Table 1). In patients with LSILs, the average numbers were 2.44 (range 1-4) and 1.88 (range 1-4), respectively; in patients with HSILs, the average numbers were 3.3 (range 2-5) and 3.5 (range 2-5), respectively; and in patients with CC, the average numbers were 2.68 (range 2-4) and 4.2 (range 3-6), respectively (Table 2). All cervical samples from patients with SILs were positive for HR-HPV. The most frequent genotypes were HPV-16 (22/49, 44%), HPV-45 and HPV-52 (7/49, 14%), HPV-18 and HPV-33 (6/49, 12%), and HPV-53 (3/49, 6%) (Table 2). Likewise, 25/49 patients had coinfection with two or more HPV genotypes, 8/18 (44.4%) of which were in patients with LSILs, 7/12 (58%) were in patients with HSILs, and 10/19 (52.6%) were in patients with CC. Patients with HSILs or CC also reported significantly greater numbers of sexual partners (averages of 3.3 and 2.6, respectively) and pregnancies (3.5 and 4.2, respectively) than NDs, whose averages were 2.26 and 1.13, respectively (Table 3).

### 3.2. PFP Samples from Patients with HSILs or CC Are High in Soluble CD39 and CD73

**Figure 1:**
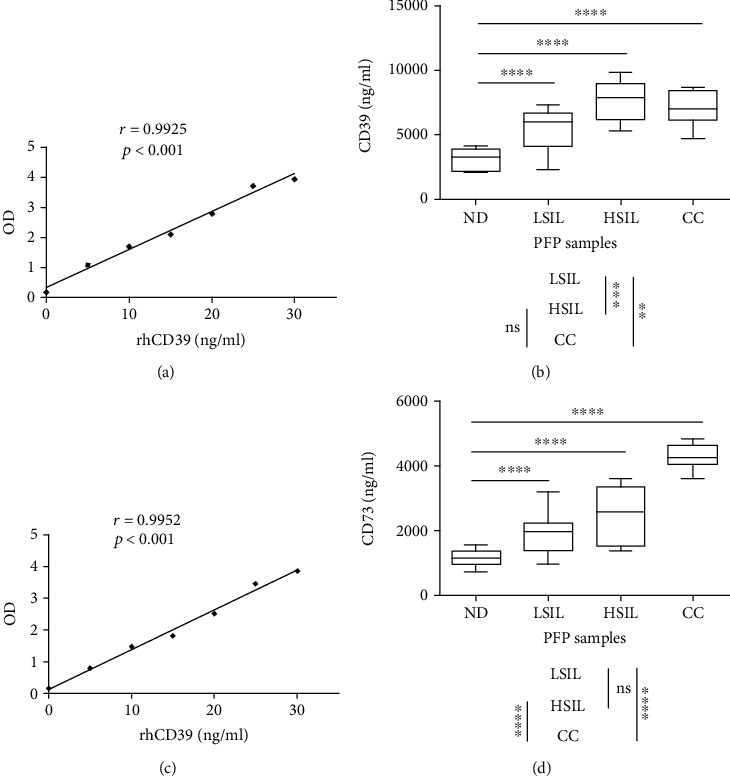
CD39 and CD73 concentrations in PFP samples from NDs and patients with LSILs, HSILs, or CC. The type curves for soluble CD39 and CD73 were established by ELISA using different concentrations (5-30 ng/ml) of human recombinant proteins CD39 (rhCD39) (a) and CD73 (rhCD73) (c), whose correlations with absorbance (optical density) were *r* = 0.9925 (*p* < 0.001) and *r* = 0.9952 (*p* < 0.001), respectively. For the detection of CD39 and CD73, the PFP samples of the NDs and women with LSILs, HSILs, or CC were diluted with PBS at ratios of 1 : 40,000 and 1 : 25,000, respectively. The data are representative of three independent experiments. The means ± SEMs of the concentrations of soluble CD39 (b) and CD73 (d) detected in the PFP of the NDs and patients with LSILs, HSILs, or CC are shown. Significant differences are indicated by ∗ (*p* < 0.05), ∗∗ (*p* < 0.001), and ∗∗∗ (*p* < 0.0001). ns: not significant.

**Figure 2:**
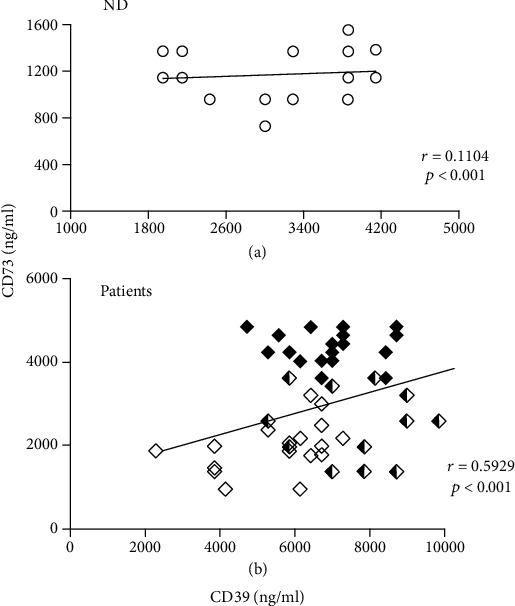
Correlations between the concentrations of CD39 and CD73 in the PFP of NDs and in the PFP of patients (LSILs, HSILs, and CC together). The correlations (*r*) between the concentrations of CD39 and CD73 in the ND PFP samples (open circles) are shown (*r* = 0.1104, *p* < 0.001) (a), as well as the correlations for patients with LSILs (white diamonds), HSILs (black and white diamonds), and CC (black diamonds) (b) (*r* = 0.5929, *p* < 0.001).

To measure soluble CD39 and CD73 in the PFP, rhCD39 curves (Figure 1(a)) and rhCD73 curves (Figure 1(c)) were established using concentrations of 5-30 ng/ml of each recombinant protein. To detect CD39 and CD73, the PFP of the NDs and of the women with LSILs, HSILs, or CC were diluted with PBS at ratios of 1 : 40,000 and 1 : 25,000, respectively. The amounts of soluble CD39 and CD73 in the PFP increased according to the degree of disease progression. Patients with LSILs, HSILs, and CC showed significantly more CD39 (averages, 6459 ± 1232 ng/ml, 8333 ± 1440 ng/ml, and 7728 ± 1198 ng/ml, respectively) than the NDs, whose average was 3839 ± 824 ng/ml (Figure 1(b)). The CD39 concentration in the PFP of patients with HSILs or CC, who showed the highest CD39, was 2.3 and 2.2 times that of the ND group, respectively (Figure 1(b)). Patients with LSILs, HSILs, or CC also showed significantly higher amounts of CD73 in the PFP (averages, 2638 ± 630 ng/ml, 3181 ± 863 ng/ml, and 5053 ± 396 ng/ml, respectively) than NDs who showed an average of 1880 ± 228 ng/ml (Figure 1(d)). The CD73 concentration in the PFP of patients with HSILs or CC, who showed the highest CD73, was 1.7 and 2.68 times that of the ND group, respectively (Figure 1(d)). Unlike NDs, who showed a low correlation between the levels of CD39 and CD73 in PFP (*r* = 0.1104, *p* < 0.001) (Figure 2(a)), the LSIL, HSIL, and CC groups had a positive correlation between the concentrations of these ectonucleotidases in PFP (*r* = 0.5929, *p* < 0.001) (Figure 2(b)).

### 3.3. PFP Samples from Patients with HSILs and CC Show a High Capacity to Generate Ado through Hydrolysis of ADP and AMP

**Figure 3:**
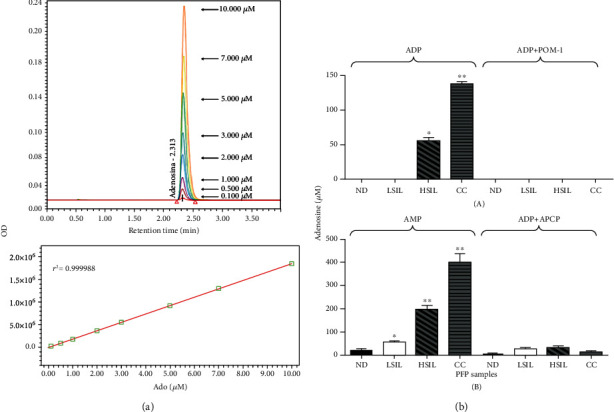
Catalytic activity of CD39 and CD73 in PFP of NDs and patients with LSILs, HSILs, or CC. Aliquots of 5 *μ*l of PFP from NDs (black bars), patients with LSILs (white bars), patients with HSILs (diagonal lines), and patients with CC (horizontal lines) were incubated in the presence of 5 mM ADP or AMP (total volume 100 *μ*l) and in the presence or absence of POM-1 or APCP, specific inhibitors of CD39 and CD73, respectively. (a) The Ado produced by hydrolysis of the nucleotides was quantified after 72 h by UPLC using standard concentrations of synthetic Ado (upper). A representative linear relationship between the Ado concentration and the optical density is shown (lower). (b) The amount of Ado produced during the incubation of PFP with ADP (upper) or AMP (lower) and in the presence or absence of POM-1 or APCP is shown. Differences in Ado concentrations were analyzed by two-way ANOVA. ^∗^*p* < 0.01, ^∗∗^*p* < 0.0001. The data represent three independent experiments.

To analyze the hydrolytic activity of the CD39 and CD73 ectonucleotidases contained in the PFP samples from NDs and patients with LSILs, HSILs, or CC, samples of 5 *μ*l of each PFP were incubated in the presence of 5 mM ADP or AMP (total volume 100 *μ*l) and in the presence or absence of POM-1 or APCP, specific inhibitors of CD39 and CD73, respectively. Aliquots of each reaction were taken at the beginning and after 72 h of incubation to evaluate Ado production through UPLC. Using different concentrations (0.1-10 *μ*M) of synthetic Ado as reference standards (Figure 3(a)), we found that the PFP derived from patients with HSIL or CC generated significantly higher amounts of Ado than the PFP of NDs or LSIL patients. The average Ado concentration produced by the ND PFP when incubated in the presence of ADP or AMP was 0 or 12.63 ± 2.3 *μ*M, respectively (Figure 3(b)), and that of the patient groups was 0 or 53.9 ± 7.88 (LSIL), 52.1 ± 1.3 or 202.9 ± 13.10 (HSIL), and 143.2 ± 7.01 or 401.3 ± 22.7 *μ*M (CC), respectively (Figure 3(b)). Interestingly, the addition of 5 mM POM-I or APCP decreased the ability of PFP to hydrolyze ADP and AMP by more than 90% in all cases (Figure 3(b)).

### 3.4. The PFP of Patients with HSILs or CC Contains Highly Glycosylated CD73

**Figure 4:**
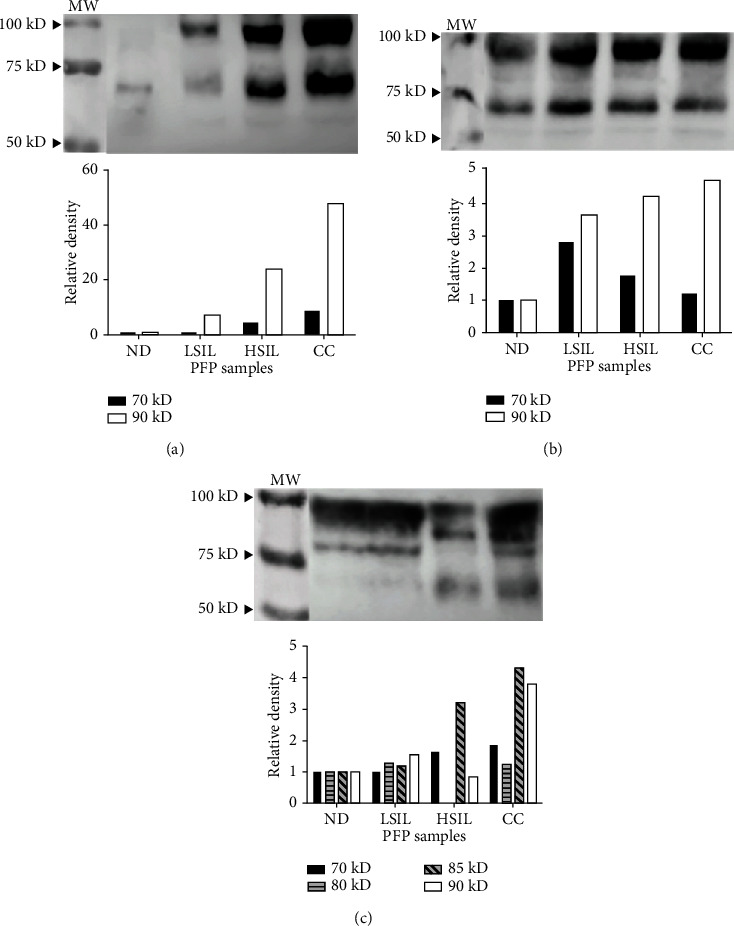
Detection of CD73 in the PFP of NDs and patients with LSILs, HSILs, or CC. Samples of 3 *μ*l of PFP (a) or 20 ng of CD73 contained in the PFP (b) of the ND, LSIL, HSIL, and CC groups were analyzed by Western blot using the anti-CD73 antibody. The densities of the 70 kD (black bars) and 90 kD (white bars) bands detected in the samples of patients with LSILs, HSILs, or CC relative to those of NDs (set to 1) are shown. (c) Twenty-nanogram samples of CD73 contained in PFP were subjected to deglycosylation using the enzymes endoglycosidase H and N-glycanase. The densities of the 70 kD (black bars), 80 kD (bars with horizontal lines), 85 kD (bars with diagonals), and 90 kD (white bars) bands detected in the LSIL, HSIL, and CC samples relative to the ND samples (set to 1) are shown. MW: molecular weight. A representative test of three independent tests is shown.

CD73 has four N-glycosylation motifs, and changes in glycosylation at one or more of these sites can alter its AMPase activity [31]. To determine whether the strong hydrolytic activity of CD73 detected in the PFP of patients with HSILs or CC was related to the degree of glycosylation, samples of 3 *μ*l of PFP (Figure 4(a)) or 20 ng of CD73 contained in the PFP (Figure 4(b)) were analyzed by Western blot using anti-CD73 antibodies. Two bands with weights of 70 kD and 90 kD approximately were revealed. Interestingly, the density of the 90 kD band detected in the PFP increased with disease progression (Figures 4(a) and 4(b)). Likewise, the 70 kD band showed higher density in the PFP of patients with HSILs or CC than in that of the LSIL or ND groups when equal volumes of PFP were analyzed (Figure 4(a)). On the other hand, when using the same amount of CD73 (20 ng), the density of the 70 kD band was similar between the PFP samples. However, a greater density of the 90 kD band was noted in the samples of the patients with LSILs, HSILs, or CC than in the ND samples (Figure 4(b)). To determine whether the 90 kD band corresponded to a highly glycosylated CD73 isoform, 20 ng samples of CD73 contained in the PFP were subjected to deglycosylation using the endoglycosidase H and N-glycanase enzymes. Enzymatic digestion of PFP samples with these enzymes resulted in products of approximately 90, 85, 80, and 70 kD according to Western blot analysis with the anti-CD73 antibody (Figure 4(c)). Samples from patients with CC showed bands corresponding to these four products. Notably, the samples of patients with HSILs and CC presented an 85 kD product, which was not detected in the samples of patients with LSILs or NDs, while an 80 kD product was detected in the samples of LSIL patients and NDs (Figure 4(c)). These results suggest that the greater hydrolytic capacity of CD73 detected in the PFP of patients with HSILs or CC may be related to a higher concentration of a highly glycosylated CD73 isoform.

### 3.5. The PFP of Patients with HSILs or CC Has Higher TGF-*β* than the PFP of Patients with LSILs and NDs

**Figure 5:**
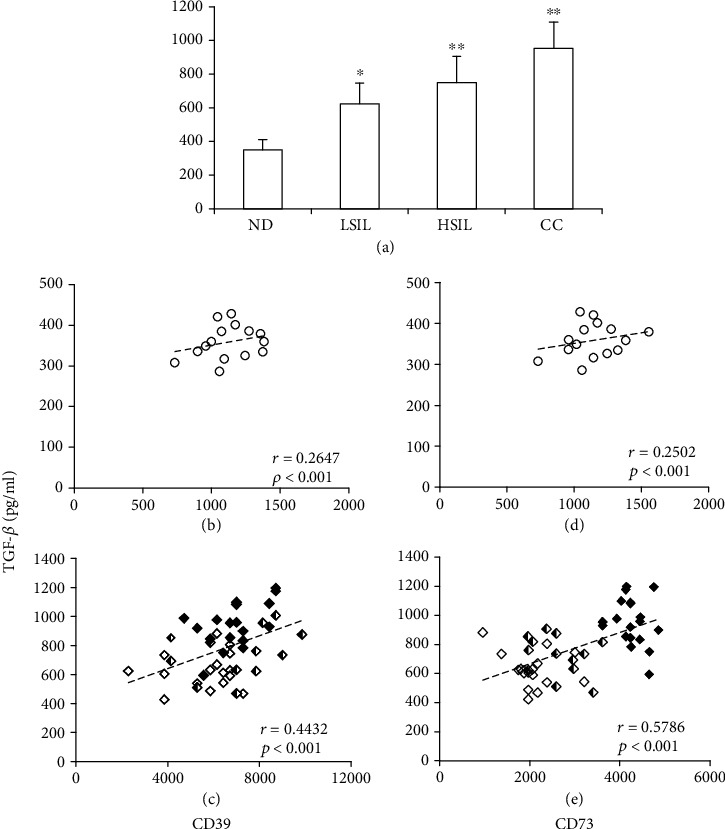
TGF-*β* concentrations in PFP samples from NDs and patients with LSILs, HSILs, or CC and their correlations with the concentrations of CD39 and CD73. (a) The TGF-*β*1 content is shown in PFP samples taken from NDs and patients with LSILs, HSILs, or CC. The data are representative of three independent experiments, and the means ± SEMs are shown. ^∗^*p* < 0.05, ^∗∗^*p* < 0.001 for the concentration of TGF-*β*1 in a patient group relative to the ND group. The correlations (*r*) between the levels of TGF-*β*1 and CD39 or CD73 in the PFP of NDs were *r* = 0.2647 (*p* < 0.001) (b) and *r* = 0.2502 (*p* < 0.001) (d), respectively. For patients with LSILs, HSILs, or CC, the correlations were *r* = 0.4432 (*p* < 0.001) (c) and *r* = 0.5786 (*p* < 0.001) (e), respectively. The coordinates of the TGF-*β*1 concentration with the CD39 or CD73 concentration are indicated by white circles in the ND group, white diamonds in the LSIL group, white and black diamonds in the HSIL group, and black diamonds in the CC group.

TGF-*β* plays an important role in promoting HPV infection and local suppression in HPV-associated neoplasms [32]. Likewise, the levels of TGF-*β* increase with the severity of cervical lesions, and the strong expression of this cytokine has been associated with poor survival in patients with CC [33, 34]. On the other hand, we recently reported that in patients with low-grade cervical neoplasms and persistent infection by HR-HPV, the highest level of expression of CD73 in cervical cells was associated with higher plasma TGF-*β* in relation to that found in NDs [25]. Likewise, we reported that TGF-*β* is important in inducing and maintaining the expression of CD73 in CC tumor cells [35]. Therefore, we proceeded to analyze the levels of this cytokine in the PFP of patients with LSILs, HSILs, or CC and compare these values with the ND value. We observed that patients with HSILs and CC, who showed the highest levels of CD39 and CD73, also showed the highest levels of TGF-*β*. The average concentration of TGF-*β* contained in the ND PFP was 350 ± 61 pg/ml, while that of LSIL, HSIL, and CC was 634 ± 122, 749 ± 155, and 954 ± 152 pg/ml, respectively (Figure 5(a)). In addition, we found a positive correlation between the concentrations of TGF-*β* with CD39 or CD73, in the PFP of patients with LSIL, HSIL, or CC (*r* = 0.4432, *p* < 0.001 (Figure 5(c)) and *r* = 0.5786, *p* < 0.001 (Figure 5(e)), respectively). In the PFP of the ND groups, the correlations were *r* = 0.2647 (*p* < 0.001) (Figure 5(b)) and *r* = 0.2502 (*p* < 0.001) (Figure 5(d)), respectively.

## 4. Discussion

In the last 10 years, cancer research has especially focused on the role played by Ado-, CD39-, and CD73-producing ectoenzymes in immunomodulation and evasion of the antitumor immune response. In fact, inhibition of the adenosinergic pathway in the tumor microenvironment has been proposed as an indispensable alternative in oncological therapy [36, 37]. However, large gaps in knowledge prevent the development of effective Ado-based therapies, such as the roles of redundant pathways that control ATP and Ado levels. Thus, the concentrations and activity of circulating ectoenzymes in the bloodstream of cancer patients may be useful as biomarkers of disease progression and to devise therapeutic strategies [38].

Our working group recently reported higher expression levels of CD39 and CD73 in cells obtained from cervical samples of patients with CINI positive for HPV-16 than in cells from HPV-16-negative samples and ND samples, which correlated with the presence of higher levels of CD39 and soluble CD73 in cervical mucus with the capacity to produce Ado through hydrolysis of ATP and AMP [25]. However, to determine whether adenosinergic activity is associated with the degree of disease progression, here, we analyzed the concentrations and activity of CD39 and CD73 in the PFP of patients with LSILs, HSILs, or CC and compared them with those of NDs. Interestingly, we found that the concentrations of CD39 and CD73 in PFP increased with the degree of disease progression. The PFP of patients with HSILs or CC showed a CD39 content 2.3 and 2.2 times that of the NDs, respectively, and a CD73 content 1.7 and 2.68 times that of the NDs. The concentrations of CD39 and CD73 in the PFP of these patients were associated with a high capacity to generate Ado from the hydrolysis of ADP and AMP. ADPase and AMPase activities were reduced by the addition of POM-1 and APCP, specific inhibitors of CD39 and CD73, respectively, suggesting that during the development of CC, alteration of nucleotide metabolism is promoted to generate increased levels of circulating Ado through the activity of these ectonucleotidases. ATPase/ADPase and AMPase activities mediated by CD39 and CD73, respectively, in the body fluids of patients with inflammatory diseases and cancer have been recently reported [39, 40]. In the context of cancer, increased expression and activity of CD39 and CD73 in tissues and/or biological fluids can lead to high levels of Ado that potently suppress the T cell-mediated antitumor immune response and promote tumor progression through stimulation of ARs [7, 41]. In fact, higher serum CD73 in patients with metastatic melanoma has been associated with lower effectiveness of nivolumab-based immunotherapy [24].

The increase in soluble CD73 found in PFP samples from patients with HSILs or CC in our study is consistent with reports of increased hydrolytic activity of AMP in the plasma of patients with advanced cancer [22, 42]. Interestingly, the increased AMPase activity found in the PFP of patients with HSILs or CC was also associated with a high concentration of a highly glycosylated 90 kD protein as revealed by Western blot assays using the anti-CD73 antibody. CD73 has four consensus N-glycosylation motifs, ^53^NAS, ^311^NSS, ^333^NYS, and ^403^NGT, and changes in glycosylation in one or more of these sites can alter the hydrolytic activity of CD73 because the three of them (N311, N333, and N403) are found in the C-terminal catalytic domain of the molecule [31]. In pathological states, the CD73 protein can undergo posttranslational changes, generating different isoforms with catalytically different properties. For example, the increase in AMPase activity in muscular dystrophy has been associated with a higher concentration of an active form of CD73 (72 kD) than an inactive form (62 kD) [43]. On the other hand, in hepatocellular carcinoma, altered CD73 glycosylation is associated with attenuated AMPase activity due to greater production of an isoform that is 50 amino acids shorter than the complete protein [44, 45]. We found that enzymatic digestion of the PFP samples using endoglycosidase H and N-glycanase resulted in products of 90, 85, 80, and 70 kD, which were observed mainly in the PFP samples of patients with HSILs or CC, suggesting that the highest degree of CD73 glycosylation in the plasma of these patients was associated with a higher AMPase capacity. In addition, the high level of CD39 and high glycosylation of CD73 in the PFP of these patients suggest that both ectonucleotidases act in a coordinated manner to generate an immunosuppressive environment through the generation of Ado, as has been proposed in other cancers [22, 42]. TGF-*β* increases the levels of CD39 and CD73 in activated T cells and myeloid suppressor cells [13]. The expression of TGF-*β*1 in CC has been directly correlated with the degree of disease progression [34] and with the expression of the HR-HPV E6 and E7 oncogenes, which induce activation of the human TGF-*β*1 promoter by recognizing the Sp1 sequence [46]. We previously reported that CC tumor cells infected with HR-HPV constitutively produce TGF-*β*, which is important for inducing and maintaining CD73 expression. We also demonstrated that Ado generated by the enzymatic activity of CD73 induced the production of TGF-*β* in tumor cells by interacting with A2AR and A2BR, suggesting an important connection between the adenosinergic pathway and the production of TGF-*β* in cells infected with HPV [35]. In this study, we found a significantly higher level of TGF-*β* in the PFP of patients with LSILs, HSILs, or CC than in that of NDs. In fact, a positive correlation was observed between the TGF-*β* concentration of these patients and the CD39 and CD73 concentrations detected in PFP. In addition, we observed that patients with HSILs or CC, who presented the highest plasma concentrations of TGF-*β*, CD39, and CD73, showed the highest numbers of sexual partners (averages of 3.3 and 2.6, respectively) and pregnancies (averages of 3.5 and 4.2, respectively) in contrast to numbers of 2.2 and 1.1, respectively, in the ND group. A strong correlation was also observed between the number of sexual partners and CD39, as well as between the number of pregnancies and the expression of CD39 and CD73, in patients with LSILs, HSILs, or CC (Table 4), which is consistent with previous reports stating that more sexual partners and pregnancies are the main risk factors associated with persistent infection by HR-HPV and the development of cervical dysplasia and CC [47, 48].

Therefore, the results obtained in this study suggest that the production of TGF-*β* associated with persistent infection by HR-HPV may be an important factor inducing and maintaining the expression of the CD39 and CD73 ectonucleotidases during the development of CC. Considering the important role of the adenosinergic pathway in the suppression of the antitumor immune response through Ado generation [13] and that the presence of high concentrations of extracellular nucleotides in the TME of CC seems to interfere with the regulation, proliferation, differentiation, and apoptosis of cancer cells of the cervix [49], the concentrations and activity of CD39 and CD73 in the plasma of patients with CC may be valuable biomarkers of disease progression and may direct the choice of clinical treatment for these patients.

## 5. Conclusions

This study provides the first evidence that the concentrations of the PFP-soluble CD39 and CD73 ectonucleotidases in patients in different stages of CC development positively correlated with disease progression and the capacity to generate Ado from the hydrolysis of ADP and AMP. The greater AMPase activity found in the PFP of patients with HSILs or CC was associated with a high concentration of a highly glycosylated 90 kD CD73 isoform. The level of TGF-*β* in the PFP of patients with LSILs, HSILs, or CC was significantly higher than that of NDs and showed positive correlations with the levels of CD39 and CD73. These results suggest that the production of TGF-*β* associated with persistent infection by HR-HPV, which is present in more than 99% of CC cases [1, 50], is a factor that promotes the expression of CD39 and CD73 to favor CC progression through Ado generation.

## Figures and Tables

**Table 1:** Clinical data of normal donors.

Cervical sample number	HPV genotypes	Age (years)	Number of sexual partners	Number of pregnancies
1	—	32	2	2
2	—	31	4	0
3	—	27	2	1
4	—	28	1	1
5	—	37	2	2
6	—	28	3	0
7	—	32	2	1
8	—	26	1	0
9	—	41	3	3
10	—	25	4	0
11	—	31	2	0
12	—	22	1	0
13	—	29	2	0
14	—	41	3	2
15	—	39	2	3
Averages	—	31.2	2.26	1

**Table 2:** Clinical data of patients with different cervical squamous cell abnormalities.

Cervical sample number	HPV genotypes	Age (years)	Number of sexual partners	Number of pregnancies
LSIL				

1	16, 58, 61	36	2	1
2	53	29	2	2
3	16, 33, 35, 52, 58	43	3	2
4	33, 71	32	3	2
5	16, 54	25	3	3
6	67	39	3	3
7	16	28	4	2
8	16, 33	43	2	4
9	66	27	3	2
10	73, 83	28	2	2
11	84	29	3	2
12	89	31	1	2
13	59, 66, 73	21	2	1
14	59	34	2	1
15	40, 52, 53, 66, 70	24	2	1
16	53	40	3	2
17	83	41	2	1
18	16	40	2	1
Averages	—	32.7	2.44	1.88

HSIL				

1	16	36	5	3
2	51, 52	29	3	4
3	45, 33	37	4	3
4	16	42	2	4
5	56, 66	44	3	2
6	16, 39	41	3	5
7	18	39	4	3
8	16	46	2	5
9	16, 45	33	3	2
10	18, 33, 45, 52	27	3	4
11	18, 53	30	4	4
12	16	34	4	3
Averages	—	36.5	3.3	3.5

SCC				

1	45, 84	42	3	4
2	33	53	2	3
3	16	39	4	4
4	16	49	2	5
5	16, 52	57	2	6
6	31	48	4	3
7	69, 71, 81, 84	62	3	4
8	39, 68	53	2	2
9	16	45	4	4
10	16, 18	38	3	5
11	16	46	3	6
12	45	40	2	5
13	45, 72	42	2	6
14	16, 62	52	2	4
15	16, 18, 52	51	3	3
16	16	35	2	4
17	18, 45	49	3	3
18	16	55	2	5
19	16, 52	50	3	4
Averages	—	47.68	2.68	4.2

**Table 3:** Correlations between clinical data of NDs and patients with different cervical squamous cell abnormalities.

The clinical data	ND	Patients			
		LSIL	HSIL	SCC	*p* values
Age (years)	31.26 ± 5.86	32.77 ± 6.94	36.5 ± 6.11^a^	47.68 ± 7.08^b,c,d^	a < 0.03 vs. ND b < 0.0001 vs. ND c < 0.0001 vs. LSIL d < 0.0001 vs. HSIL

Number of sexual partners	2.26 ± 0.96	2.44 ± 0.7	3.3 ± 0.88^e,f^	2.6 ± 0.75^g^	e < 0.006 vs. ND f < 0.005 vs. LSIL g < 0.03 vs. HSIL

Number of pregnancies	1.13 ± 0.2	1.88 ± 0.83^h^	3.5 ± 1^i,j^	4.2 ± 1.1^k,l^	h < 0.01 vs. ND i < 0.0001 vs. ND j <0.0001 vs. LSIL k < 0.0001 vs. ND l < 0.001 vs. LSIL

ND: normal donor; LSIL: low-grade squamous intraepithelial lesion; HSIL: high-grade squamous intraepithelial lesion; SCC: squamous cell carcinoma. *p* values were calculated using the Wilcoxon signed-rank test and Student's *t*-test.

**Table 4:** Correlation analysis of the clinical data of normal donors and patients with SIL and the plasmatic contents of the nucleotidases CD39 and CD73.

Nucleotidase	Normal donors			SIL patients			*p* values
	Age (years)	Number of sexual partners	Number of pregnancies	Age (years)	Number of sexual partners	Number of pregnancies	
CD39	-0.06017	-0.2257	0.0721	0.1308	0.3312^a^	0.3512^b^	a < 0.0201 b < 0.0134 c < 0.0001 d < 0.0001
CD73	0.3028	0.2277	0.2284	0.5868^c^	0.1542	0.6387^d^	

Values of Pearson's coefficient (*r*) are shown.

## Data Availability

The data used to support the findings of this study are available from the corresponding author upon request.

## Abbreviations

Ado: Adenosine
ADP: Adenosine diphosphate
AMP: Adenosine monophosphate
APCP: 5′-(*α*,*β*-Methylene) diphosphate
ARs (A1R, A2AR, A2BR, and A3R): Adenosine receptors
ATP: Adenosine triphosphate
CC: Cervical cancer
CD39: Ectonucleoside triphosphate diphosphohydrolase-1
CD73: 5′-Nucleotidase
CINI, CINII, and CINIII: Cervical intraepithelial neoplasia grades I, II, and III, respectively
CTL: Cytotoxic T lymphocyte
HR-HPV: High-risk human papillomavirus
HSIL: High-grade squamous intraepithelial lesion
LSIL: Low-grade squamous intraepithelial lesion
NDs: Normal donors
NILM: Negative for intraepithelial lesion or malignancy
PCR: Polymerase chain reaction
PFP: Platelet-free plasma
POM-1: Sodium polyoxotungstate
TGF-*β*: Transforming growth factor-*β*
UPLC: Ultra-high-performance liquid chromatography.

## Supplementary Materials

Supplementary Materials Supplementary Figure 1: detection of the HPV L1 gene in cervical samples from NILM women. Expression of the L1 gene in cervical samples was detected by PCR as described in Section 2. The absence (left) or presence (right) of L1 is shown in samples from NILM women. As a positive control, DNA from the HeLa cell line (HPV-18^+^) was used. The expression of the *β*-actin gene in all samples was used as an internal control. The size of the fragment (bp) of each gene is indicated.
