# Supplementary figures and images for: Detection of CD39 and a Highly Glycosylated Isoform of Soluble CD73 in the Plasma of Patients with Cervical Cancer: Correlation with Disease Progression
Source: Mediators Inflamm. 2020 Dec 7;2020:1678780. doi: 10.1155/2020/1678780 (PMC7803102; doi:10.1155/2020/1678780)

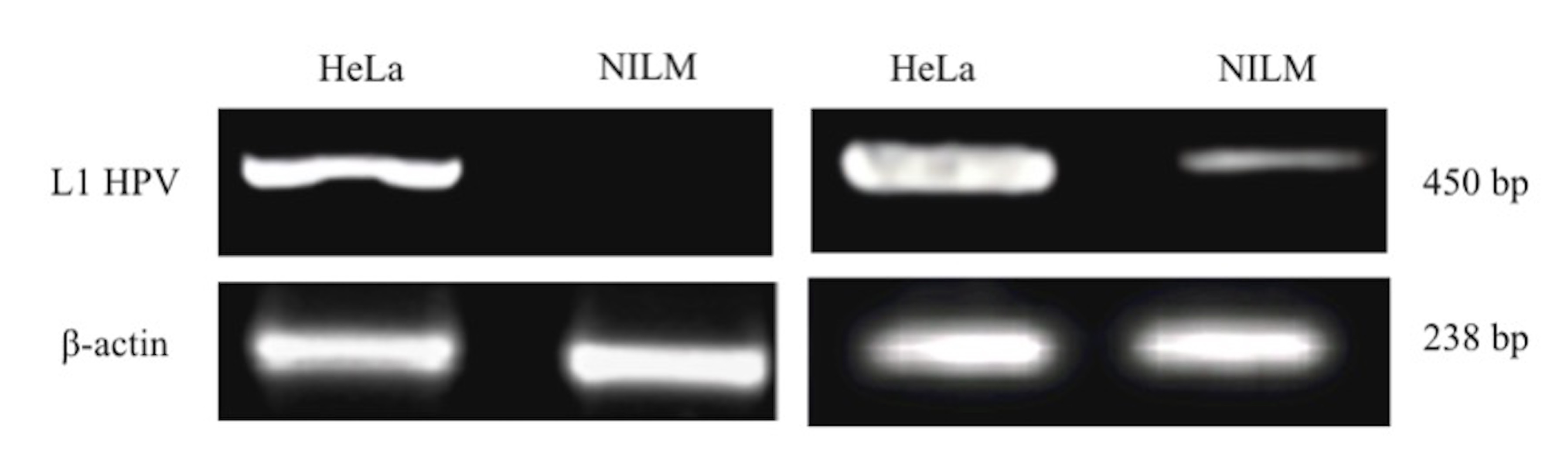

Supplement: Supplementary Materials — Supplementary Figure 1: detection of the HPV L1 gene in cervical samples from NILM women. Expression of the L1 gene in cervical samples was detected by PCR as described in Section 2. The absence (left) or presence (right) of L1 is shown in samples from NILM women. As a positive control, DNA from the HeLa cell line (HPV-18+) was used. The expression of the β-actin gene in all samples was used as an internal control. The size of the fragment (bp) of each gene is indicated. [file 1678780.f1.jpg]
